# On the Brink of the Abyss: Managing More Than 25 Liters of Blood Loss During a Cesarean Section

**DOI:** 10.7759/cureus.95589

**Published:** 2025-10-28

**Authors:** Strahil N Kotsev, Nadine B Nour, Muhammad Sharif Allah Dad, Zainab Saeed

**Affiliations:** 1 Department of Anaesthesia and Intensive Care, Latifa Hospital, Dubai Health, Dubai, ARE

**Keywords:** aortic clamp, cesarean section, massive blood transfusion, obstetric hemorrhage, surgical management of obstetrical hemorrhage

## Abstract

We present a case of placenta percreta in which the patient experienced an intraoperative blood loss exceeding 25 liters. To our knowledge, this represents the highest volume of hemorrhage reported during cesarean section (CS) in which the patient survived. Injuries of the iliac vessels, urinary bladder, and ureter had to be dealt with intraoperatively. An infrarenal aortic clamp stemmed the bleeding. We discuss the challenges presented by such cases, personalized transfusion, and the controversies surrounding the current treatment strategies.

## Introduction

The perioperative management of an abnormally adherent placenta (AAP) presents unique challenges. From a diagnostic viewpoint, the exact extent to which the placenta invades the uterine wall and/or surrounding structures is often unknown preoperatively. Both ultrasound and placental magnetic resonance imaging (MRI) can be used to ascertain the extent of invasion to a significant degree. Often, radiologists are not exposed enough to placental MRI, and reports do not describe with certainty the degree of invasion. The ultrasound has its own limitations even in the hands of experienced operators [1].

From surgical perspectives, although the approach to the cesarean section (CS) in cases of AAP is somewhat standardized, controversies do exist [2]. Conservative versus surgical management, the type and extent of surgical treatment, interventional radiologist involvement, balloon occlusion, etc., are parts of the dilemma [3,4]. The roles and responsibilities of the team members are often ill-defined. They frequently cross the narrow borders of specialties.

Personalized transfusion management, assessment of fluid responsiveness, and postoperative critical care skills are essential when dealing with such cases [5]. Organizational leadership can positively impact the outcome.

## Case presentation

The patient was 38 years old, para 8, gravida 9, with four previous CSs. She had been diagnosed with a case of AAP and was scheduled for CS at 34 weeks at the main maternity hospital in Dubai. The parturient had essential hypertension, anemia, obesity (BMI 46.6 kg/m²), and gestational diabetes mellitus. The hypertension and gestational diabetes were well controlled. There were no fetal concerns. During her previous pregnancy, methods of contraception had been discussed, but the patient and her husband refused them. Preoperative work-up was done as per the hospital policies and protocols. The members of the multidisciplinary team saw the patient on many occasions. The consultant anesthesiologist discussed the anesthesia plan with the patient. The discussions were documented, and informed consent was obtained. Standard communication channels were activated. The transfusion hematology center, operating theater, ICU, psychologist, and hospitals in the Dubai Health Authority were alerted. 

On the day of the operation, after receiving antacid prophylaxis, the patient was transferred to the operating theater (OT). An antibiotic was administered. A warm air blanket, fluid warmer, and sequential pneumatic compression device were put on. Two 8.5 Fr rapid infusion catheters (ArrowFlex®, Teleflex Inc., Wayne, PA) in big peripheral veins and an arterial line were inserted under ultrasound guidance. A central venous catheter (introducer sheath 8.5 Fr) was inserted using sonography. Ten units of packed red blood cells (PRBCs), six units of thawed plasma, and a pool of platelets were present in the operating theater at the start of the surgery. Tranexamic acid was administered. Initial cystoscopy confirmed AAP with invasion of the urinary bladder. Ureteric stents were inserted. The uterus was opened at the fundus, away from the placenta and the previous scars, and the baby was delivered with an excellent Appearance, Pulse, Grimace, Activity, and Respiration (Apgar) score. The obstetricians and the oncogynecologist decided to manage the case conservatively. The uterus was closed, leaving the placenta inside. After closing the uterine wall, torrential bleeding ensued, and a hysterectomy had to be done. The massive transfusion protocol was activated. During the hysterectomy, inadvertent damage to the bladder, right iliac vessels, and left ureter occurred. The patient was bleeding from the severed left ureter, injured right iliac vessels, and the remaining part of the urinary bladder. The vascular surgeons were called in. An aortic cross-clamp was suggested by the anesthesiologist as the rate of transfusion was not able to match the speed of bleeding in spite of using two Level One® rapid infusion devices (ICU Medical, Inc., San Clemente, CA). Two suctions were used in the operative field. Manual compression of the aorta by one of the assistants had been applied until a temporary infraceliac aortic clamp was put in by the vascular surgeons using Mattox's maneuver. The patient's hemodynamics improved within minutes after the application of the aortic cross-clamp. The iliac vessel injuries were dealt with. The bladder had to be repaired, and the ureter reimplanted by the urologists. The bleeding was stopped.

An abdominal pack was left in the pelvis to compress the oozing points, and the skin of the abdomen was loosely closed (damage control). 

Doses of antibiotics, calcium, and recombinant factor VIIa were given intraoperatively. Rapid thromboelastography (RapidTEG®, Haemonetics Corporation, Boston, MA) and arterial blood gas analyses were obtained during the operation to help guide the transfusion. Laboratory results were obtained as clinically indicated (Table 1).

**Table 1:** Relevant laboratory results of the patient

Test	Reference range	Units	Patient values obtained at intervals during the nine-hour surgery						
Hemoglobin	11-15	g/dl	10.5	10.5	6.2	6.3	9.9	9.1	10.9
Hematocrit	36-45	%	32.9	31.9	18.7	19	28.7	26.6	31.1
Platelets	150-400,000	mcl	130	142	54	47	166	178	165
Prothrombin time	14-15	seconds	13.3	13.7	15.4	12.4	12.8	13.8	14
International normalized ratio	1.0	-	1.08	1.11	1.28	0.97	1.01	1.11	1.13
Activated partial thromboplastin time	28-41	seconds	31.8	40.9	41.3	42.1	39.5	36	31.9
Fibrinogen	200-600	mg/dl	400	185	146	228	221	291	289
Lactate	0.5-1.8	mmol/l	1.8	2.2	4.5	3.6	3.4	2.8	2.5

**Figure 1:**
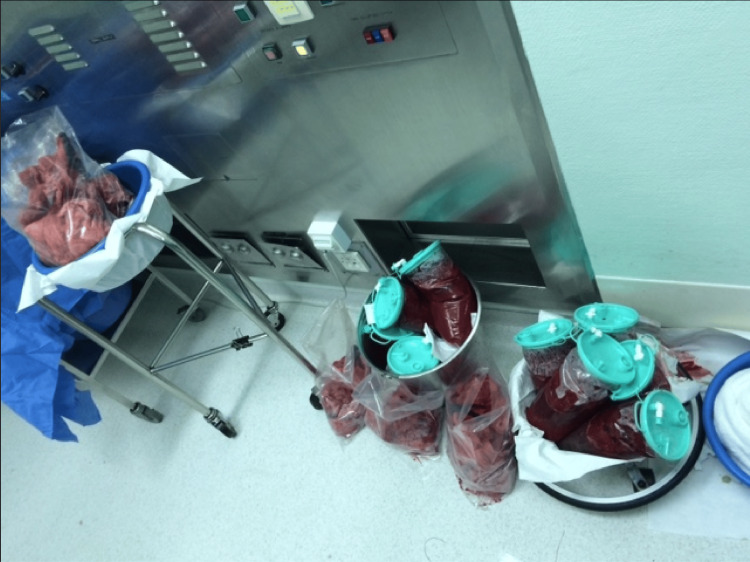
Part of the 25 liters of blood lost by the patient during the cesarean section

Throughout the nine hours of surgery, the coagulation status of the patient, temperature, and urine output were within acceptable ranges. Intraoperatively, the patient sustained an estimated blood loss of more than 25 liters. The circulating blood volume (CBV) in an average adult is around 5 liters. Pregnant patients do have increased CBV of 30%-40%, mainly due to increased plasma volume [6]. Our patient was 126 kg. Intraoperatively, she sustained an estimated blood loss of more than 25 liters. It constituted several times the exchange of her CBV (Figure 1). 

Throughout the surgery, the patient did not require inotropic support, as focused echo examinations revealed a hyperdynamic left ventricle.

The patient was ventilated overnight (lung protective ventilation strategy). Sedatives, analgesics, and neuromuscular blocking agents were used during the first 16 hours in the ICU, where an anesthesiologist managed the patient. The intra-abdominal pressure did not reach critical values. Frequent echocardiography examinations through standard and atypical windows were performed perioperatively. Lung sonography, ultrasound assessment of fluid responsiveness, and laboratory indices guided the transfusion therapy. The next day, the pelvic pack was removed, the abdomen was inspected for bleeding and closed, and the patient was extubated. The edema resulting from the massive transfusion resolved in the following days. On postoperative day 5, the patient was transferred to the ward. 

Forty units of PRBCs, 29 units of fresh frozen plasma, 48 cryoprecipitates, 12 platelet pools, and several liters of crystalloids were transfused during the course of the events. Debriefing was done.

## Discussion

The following points merit a brief discussion.

Patient and family education

Risk avoidance is better than risk management. Although the obstetric team had addressed the issue of contraception, the patient and her husband refused it. Better counseling could have led to better results.

Conservative management of AAP versus hysterectomy

Although well described in the literature, conservative management of the placenta is not free of risks [7,8]. The placenta does not invade the uterine wall and/or surrounding structures evenly. There are areas of normal placentation, too. When the placenta starts to separate from the areas of normal adherence, torrential bleeding can ensue. It can happen at a time when surgical and other expertise to deal with it is not immediately available. The risk of infection is a present danger, as the left placenta is a medium for bacterial growth. Our institutional experience with 16 cases of attempted conservative management of AAP has been a negative one so far. Life-threatening obstetric hemorrhage and infection have been the main issues. In most cases, the patients did have prolonged hospital stays.

In the author's opinion, rework is expensive and wastes time, energy, and human resources. In many of the above cases, it has created unacceptable risks for the patients.

Prophylactic occlusion of the aorta or a cross clamp 

Prophylactic temporary occlusion of the aorta or infra-celiac cross clamp might be considered in cases where the placenta invades the surrounding organs and the potential for massive obstetric hemorrhage is high. An infraceliac aortic clamp is not associated with a significant increase in myocardial wall tension. It does help to stem the bleeding from the uterus, bladder, ureter, and iliac vessels [9]. Infraceliac aortic clamping increases the blood pressure by the mechanical effect of the clamp, the release of vasoactive substances, and other mechanisms. Resuscitative endovascular balloon occlusion of the aorta might play a role in such cases, too [10].

Ligation of the internal iliac artery in case of hysterectomy

One should not venture into uncharted waters unless he/she is well prepared for it. Ligation of the internal iliac artery is not risk-free. It might be a step towards doom. Some of the potential complications associated with it are injuries of the internal iliac vein (thin wall, easily retracting, and difficult to repair), bleeding, ligation of the external iliac artery, ischemia of the leg, etc. [11, 12]. 

Management of massive transfusion: less is more

Personalized hemodynamic management, using ultrasound indices for fluid responsiveness, frequent echocardiography, lung ultrasound, and laboratory indices of microcirculation, helps guide transfusion [13,14]. It can also reduce potential complications associated with massive transfusion-transfusion-related lung injury (TRALI), transfusion-associated circulatory overload (TACO), bowel edema, and abdominal compartment syndrome (ACS).

Point-of-care devices to follow the trend in coagulation status can reduce the amount of blood products transfused and guide therapy. Parturients are hypercoagulable, and values for the TEG® in pregnancy have been reported [15]. The hypercoagulable status must be maintained during such operations.

The initial resuscitation must be guided by clinical indices. Lyophilized or thawed AB negative plasma, prothrombin complex concentrate, and whole blood do play a role in the pre-hospital and emergency management of bleeding patients [16, 17].

Blood component management later on can be guided by viscoelastic studies and/or laboratory indices [18]. Current guidelines recommend the use of tranexamic acid and cell-saver. The clinical impact of it on meaningful outcomes might not be so great [19, 20].

Better organization usually leads to better results. Not all of the people present during the operation added value. The law of diminishing returns apparently is true. Adopting lean thinking and the principles of Lean Six Sigma and business process management can greatly improve performance. Human resource management can be better. Processes can be simplified, streamlined, and standardized [21]. 

Organizational complexities can be delayered, keeping the patient and the desired outcomes in focus. And only then can the people be trained to perform as a team in an optimized process. Automating wrong processes and training people in courses and simulations to follow them is automating failure. Organizational leadership must provide an environment conducive to patient safety.

## Conclusions

We have described the successful management of a parturient with placenta percreta. Injuries of the urinary bladder, iliac vessels, and ureter had occurred, and an aortic cross clamp was deployed to arrest the bleeding. The patient suffered an enormous blood loss of more than 25 liters. Occlusion of the aorta in case of massive obstetric hemorrhage does play a role. It must be done preemptively if possible. Proactive management trumps reactive management. Due in part to personalized transfusion, point-of-care technology, and rapid decision-making by the vascular surgeons, the patient has made an uneventful recovery.
